# Supplementary material for: Transmission dynamics of MERS-CoV in a transgenic human DPP4 mouse model
Source: Npj Viruses. 2024 Aug 21;2:36. doi: 10.1038/s44298-024-00048-y (PMC11721671; doi:10.1038/s44298-024-00048-y)
Supplement: Supplementary file 1 — Supplementary Data [file 44298_2024_48_MOESM1_ESM.docx]

**Transmission dynamics of MERS-CoV in a transgenic human DPP4 mouse model**

**Supplementary Data**

Neeltje van Doremalen PhD^1^, Trenton Bushmaker MSc^1^, Robert J. Fischer PhD^1^, Atsushi Okumura DVM^2^, Dania M. Figueroa Acosta, BA^1,4^, Rebekah J. McMinn BSc^1^, Michael Letko PhD^1^, Dana Scott DVM^3^, Greg Saturday DVM, DACVP, DABT^3^, Vincent J. Munster PhD^1*^

1 Laboratory of Virology, Division of Intramural Research, National Institute of Allergy and Infectious Diseases, National Institutes of Health, Hamilton, MT, USA

2 Paul G. Allen School for Global Health, Washington State University, Pullman, WA, USA

3 Rocky Mountain Veterinary Branch, Division of Intramural Research, National Institute of Allergy

and Infectious Diseases, National Institutes of Health, Hamilton, MT, USA

4 Current affiliation: Division of Infectious Diseases, Department of Medicine, Icahn School of Medicine at Mount Sinai, New York, NY, USA

*Corresponding author: Vincent Munster, email: [vincent.munster@nih.gov](mailto:Vincent.munster@nih.gov)

**
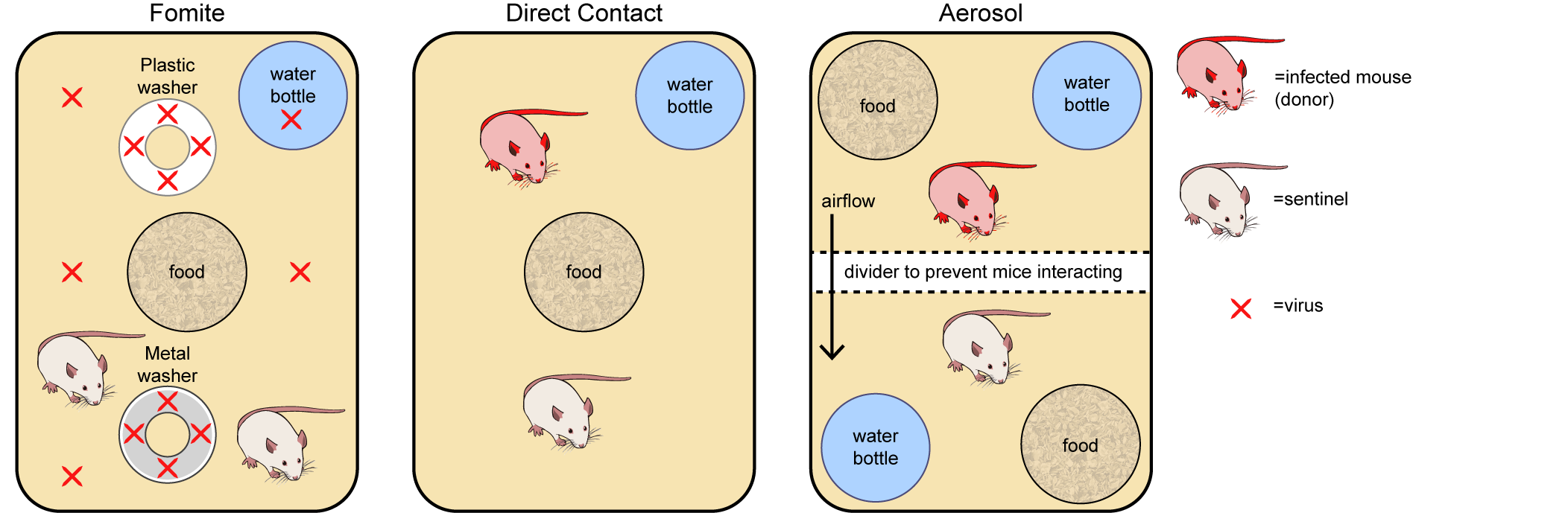
**

**Figure S1. Cage setup during transmission experiments.** Depicted are cage setups for fomite, direct contact, and airborne transmission.
